# The Home Treatment of Disease

**Published:** 1898-08-06

**Authors:** 


### THE HOME TREATMENT OF DISEASE.

In opening the section of Diseases of Children the president, Dr. Joseph Bell, of Edinburgh, expressed the opinion that there was a risk of overdoing what might be called the driving of sick and hurt children into hospital, to the exclusion of home treatment. That might be considered a foolish idea, but the general practitioner all over the country was gradually beginning to see that hospitals were being used by patients who had no right whatever to public charity, to the grave detriment of the independence of the middle class, who were already too well disposed to get all they could for nothing, and also impoverishing the practitioner. The very poor, who had dirty homes, and who starved their children, might have a sort of reason when they brought their children to light, air, and food; but the great middle class should be reminded of their responsibilities. There was no reason whatever why a large number of operations which crowded the wards should not be done at home. Mastoid antrum cases, tuberculous glands and joints, radical cures of hernia, phimosis, and the like—why should hospital surgeons slave at doing these for nothing for patients whose relations were often much richer than the surgeon? Oh, the answer was, they could not keep the wound aseptic. Well, if they could not keep a child aseptic in a decent home, they were not much worth; and till the State paid the doctor, or public charity supported him as well as the palatial hospital, some patients should still be left in their homes. Besides, for a small child, could any nursing in hospital, however good—and they knew how good it generally was—make up to the child for removal from its mother; or, in dying cases, comfort the mother who had no longer the child to nurse, but only was allowed an hour by its bedside in a long ward? In conclusion, he asked his hearers to think of these things, for hospital surgeons were apt to forget the family tie as well as the family practitioner.

